# 2-(2,4-Dichloro­phen­oxy)-1-(1*H*-pyrazol-1-yl)ethanone

**DOI:** 10.1107/S1600536810035087

**Published:** 2010-09-04

**Authors:** Aisha Karamat, M. Nawaz Tahir, Misbahul Ain Khan, Abdul Qayyum Ather

**Affiliations:** a Institute of Chemistry, University of the Punjab, Lahore, Pakistan; b Department of Physics, University of Sargodha, Sargodha, Pakistan; c Department of Chemistry, Islamia University, Bahawalpur, Pakistan; d Applied Chemistry Research Center, PCSIR Laboratories Complex, Lahore 54600, Pakistan

## Abstract

In the title compound, C_11_H_8_Cl_2_N_2_O_2_, the 2,4-dichloro­phen­oxy and 1*H*-pyrazole groups are almost planar [r.m.s. deviations of 0.0157 and 0.0008 Å, respectively] and are oriented at a dihedral angle of 64.17 (5)° with respect to one another. In the crystal, the mol­ecules are stabilized in the form of dimers due to inversion-related C—H⋯O hydrogen bonds, with *R*_2_^2^(10) ring motifs.

## Related literature

Aryl­oxyacetic acid and its various derivatives are used as herbicides and pesticides, see: Crafts (1957 ▶). For our work on the synthesis of heterocyclic compounds, see: Khan *et al.* (2009 ▶). For a related structure, see: Wang *et al.* (2009 ▶). For graph-set notation, see: Bernstein *et al.* (1995 ▶).
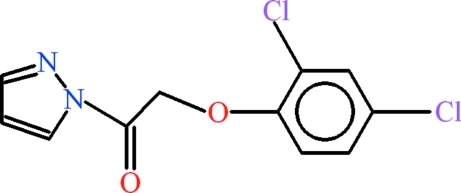


## Experimental

### 

#### Crystal data

C_11_H_8_Cl_2_N_2_O_2_
*M* *_r_* = 271.09
Triclinic, 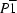

*a* = 4.2030 (1) Å
*b* = 10.3074 (3) Å
*c* = 13.4966 (4) Å
α = 87.510 (2)°
β = 83.774 (1)°
γ = 88.335 (1)°
*V* = 580.53 (3) Å^3^
*Z* = 2
Mo *K*α radiation
μ = 0.55 mm^−1^
*T* = 296 K
0.30 × 0.22 × 0.18 mm

#### Data collection

Bruker Kappa APEXII CCD diffractometer
Absorption correction: multi-scan (*SADABS*; Bruker, 2005 ▶) *T*_min_ = 0.982, *T*_max_ = 0.988
10353 measured reflections
2861 independent reflections
2232 reflections with *I* > 2σ(*I*)
*R*_int_ = 0.025

#### Refinement

*R*[*F* ^2^ > 2σ(*F* ^2^)] = 0.035
*wR*(*F* ^2^) = 0.093
*S* = 1.04
2861 reflections
154 parameters
H-atom parameters constrained
Δρ_max_ = 0.25 e Å^−3^
Δρ_min_ = −0.29 e Å^−3^

### 

Data collection: *APEX2* (Bruker, 2009 ▶); cell refinement: *SAINT* (Bruker, 2009 ▶); data reduction: *SAINT*; program(s) used to solve structure: *SHELXS97* (Sheldrick, 2008 ▶); program(s) used to refine structure: *SHELXL97* (Sheldrick, 2008 ▶); molecular graphics: *ORTEP-3 for Windows* (Farrugia, 1997 ▶) and *PLATON* (Spek, 2009 ▶); software used to prepare material for publication: *WinGX* (Farrugia, 1999 ▶) and *PLATON*.

## Supplementary Material

Crystal structure: contains datablocks global, I. DOI: 10.1107/S1600536810035087/si2292sup1.cif

Structure factors: contains datablocks I. DOI: 10.1107/S1600536810035087/si2292Isup2.hkl

Additional supplementary materials:  crystallographic information; 3D view; checkCIF report

## Figures and Tables

**Table 1:** Hydrogen-bond geometry (Å, °)

*D*—H⋯*A*	*D*—H	H⋯*A*	*D*⋯*A*	*D*—H⋯*A*
C11—H11⋯O2^i^	0.93	2.42	3.339 (2)	170

Symmetry code: (i) 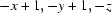.

**Fig. 1.**
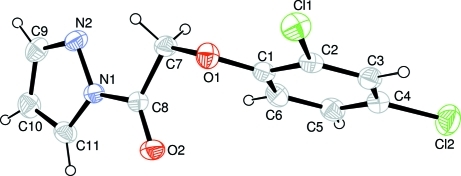
View of the title compound with the atom numbering scheme. The thermal ellipsoids are drawn at the 50% probability level. H-atoms are shown as small spheres of arbitrary radii.

**Fig. 2.**
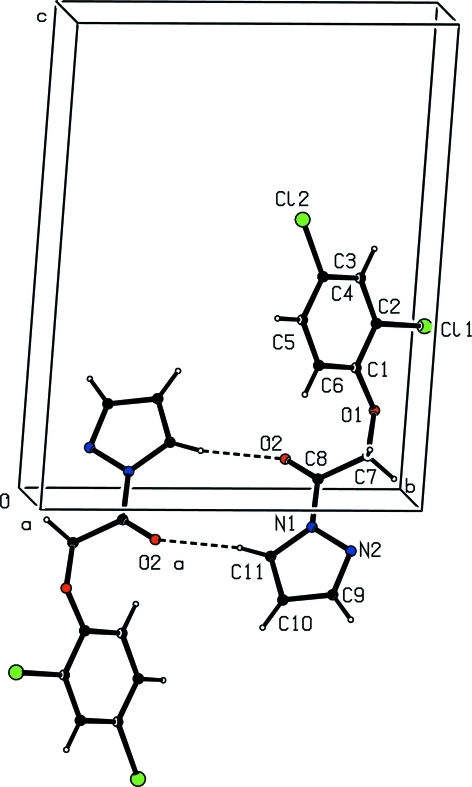
Packing section of the title compound (PLATON: Spek, 2009) showing that molecules are stabilized in the form of dimers.

## supplementary crystallographic information

### Comment

Aryloxyacetic acid and its various derivatives are used as herbicides and pesticides (Crafts, 1957). During our research on the synthesis of heterocyclic compounds in our laboratories (Khan *et al.*, 2009), we have isolated the title compound (I, Fig. 1).

The crystal structure of 5-(2,4-dichlorophenoxymethyl)-1,3,4-thiadiazol-2-amine has been published (Wang *et al.*, 2009) which is related to the title compound.

In the title compound, 2,4-dichlorophenoxy group A (O1/C1—C6/CL1/CL2) and 1*H*-pyrazole group B (N1/N2/C9–C11) are planar with r. m. s. deviations of 0.0157 and 0.0008 Å, respectively. The dihedral angle between A/B is 64.17 (5)°. The central group C (C7/C8/O2) is of course planar. The dihedral angle between A/C and B/C is 69.23 (8) and 5.07 (25)°, respectively. The molecules are stabilized in the form of dimers (Table 1, Fig. 2) due to inversion related C—H···O type of H-bondings with *R*_2_^2^(10) ring motifs (Bernstein *et al.*, 1995).

### Experimental

A mixture of 2,4-dichlorophenoxyacetic acid (0.5 g; 2.25 mmole) and 1 ml of thionyl chloride was heated under reflux for 1 h. Then an excess of pyrazole (0.5 g) in 5 ml of chloroform was added to the refluxing mixture and heated for a further period of 1.5 h. The solvents were removed and the residue dissolved in chloroform and washed with saturated sodium bicarbonate, dried and let crystallize to give pale brown prisms of (I).

Yield, 84%.

### Refinement

The H-atoms were positioned geometrically (C–H = 0.93–0.97 Å) and were included in the refinement in the riding model approximation, with *U*_iso_(H) = x*U*_eq_(C), where x = 1.2 for all H-atoms.

### Crystal data

**Table:**

C_11_H_8_Cl_2_N_2_O_2_	*Z* = 2
*M**_r_* = 271.09	*F*(000) = 276
Triclinic, *P*1	*D*_x_ = 1.551 Mg m^−^^3^
Hall symbol: -P 1	Mo *K*α radiation, λ = 0.71073 Å
*a* = 4.2030 (1) Å	Cell parameters from 2920 reflections
*b* = 10.3074 (3) Å	θ = 2.6–27.9°
*c* = 13.4966 (4) Å	µ = 0.55 mm^−^^1^
α = 87.510 (2)°	*T* = 296 K
β = 83.774 (1)°	Prismatic, pale brown
γ = 88.335 (1)°	0.30 × 0.22 × 0.18 mm
*V* = 580.53 (3) Å^3^	

### Data collection

**Table:**

Bruker Kappa APEXII CCD diffractometer	2861 independent reflections
Radiation source: fine-focus sealed tube	2232 reflections with *I* > 2σ(*I*)
graphite	*R*_int_ = 0.025
Detector resolution: 7.50 pixels mm^-1^	θ_max_ = 28.4°, θ_min_ = 2.0°
ω scans	*h* = −5→5
Absorption correction: multi-scan (*SADABS*; Bruker, 2005)	*k* = −13→13
*T*_min_ = 0.982, *T*_max_ = 0.988	*l* = −17→17
10353 measured reflections	

### Refinement

**Table:**

Refinement on *F*^2^	Primary atom site location: structure-invariant direct methods
Least-squares matrix: full	Secondary atom site location: difference Fourier map
*R*[*F*^2^ > 2σ(*F*^2^)] = 0.035	Hydrogen site location: inferred from neighbouring sites
*wR*(*F*^2^) = 0.093	H-atom parameters constrained
*S* = 1.04	*w* = 1/[σ^2^(*F*_o_^2^) + (0.0378*P*)^2^ + 0.1536*P*] where *P* = (*F*_o_^2^ + 2*F*_c_^2^)/3
2861 reflections	(Δ/σ)_max_ = 0.001
154 parameters	Δρ_max_ = 0.25 e Å^−^^3^
0 restraints	Δρ_min_ = −0.29 e Å^−^^3^

### Special details

**Table:**

Geometry. Bond distances, angles *etc*. have been calculated using the rounded fractional coordinates. All su's are estimated from the variances of the (full) variance-covariance matrix. The cell e.s.d.'s are taken into account in the estimation of distances, angles and torsion angles
Refinement. Refinement of *F*^2^ against ALL reflections. The weighted *R*-factor *wR* and goodness of fit *S* are based on *F*^2^, conventional *R*-factors *R* are based on *F*, with *F* set to zero for negative *F*^2^. The threshold expression of *F*^2^ > σ(*F*^2^) is used only for calculating *R*-factors(gt) *etc*. and is not relevant to the choice of reflections for refinement. *R*-factors based on *F*^2^ are statistically about twice as large as those based on *F*, and *R*- factors based on ALL data will be even larger.

### Fractional atomic coordinates and isotropic or equivalent isotropic displacement parameters (Å^2^)

**Table:**

	*x*	*y*	*z*	*U*_iso_*/*U*_eq_	
Cl1	0.18429 (10)	1.01977 (4)	0.34325 (3)	0.0568 (2)	
Cl2	0.65932 (16)	0.65004 (6)	0.58253 (4)	0.0775 (2)	
O1	0.5309 (3)	0.88439 (11)	0.18509 (8)	0.0484 (4)	
O2	0.4918 (3)	0.66285 (12)	0.08285 (9)	0.0547 (4)	
N1	0.8767 (3)	0.72296 (12)	−0.03954 (9)	0.0409 (4)	
N2	1.0808 (3)	0.81744 (14)	−0.07931 (11)	0.0508 (5)	
C1	0.5754 (3)	0.82450 (15)	0.27460 (11)	0.0409 (5)	
C2	0.4121 (3)	0.87940 (15)	0.35859 (12)	0.0414 (5)	
C3	0.4354 (4)	0.82649 (16)	0.45278 (12)	0.0484 (5)	
C4	0.6248 (4)	0.71690 (17)	0.46376 (13)	0.0499 (5)	
C5	0.7924 (4)	0.66131 (17)	0.38241 (13)	0.0534 (6)	
C6	0.7676 (4)	0.71501 (17)	0.28800 (13)	0.0498 (5)	
C7	0.7588 (4)	0.86184 (16)	0.10197 (12)	0.0456 (5)	
C8	0.6884 (4)	0.74046 (15)	0.05091 (11)	0.0404 (5)	
C9	1.2064 (5)	0.77078 (19)	−0.16381 (13)	0.0589 (6)	
C10	1.0901 (5)	0.6484 (2)	−0.17990 (13)	0.0609 (7)	
C11	0.8813 (4)	0.62017 (17)	−0.09989 (13)	0.0523 (6)	
H3	0.32501	0.86416	0.50819	0.0580*	
H5	0.92191	0.58786	0.39101	0.0641*	
H6	0.88072	0.67744	0.23299	0.0597*	
H7A	0.97086	0.85358	0.12400	0.0547*	
H7B	0.75702	0.93557	0.05484	0.0547*	
H9	1.35614	0.81406	−0.20817	0.0706*	
H10	1.14575	0.59735	−0.23456	0.0731*	
H11	0.76304	0.54516	−0.08799	0.0627*	

### Atomic displacement parameters (Å^2^)

**Table:**

	*U*^11^	*U*^22^	*U*^33^	*U*^12^	*U*^13^	*U*^23^
Cl1	0.0547 (3)	0.0481 (2)	0.0649 (3)	0.0094 (2)	0.0060 (2)	−0.0084 (2)
Cl2	0.1045 (4)	0.0712 (3)	0.0552 (3)	0.0028 (3)	−0.0074 (3)	0.0102 (2)
O1	0.0497 (6)	0.0498 (7)	0.0447 (6)	0.0070 (5)	−0.0004 (5)	−0.0064 (5)
O2	0.0566 (7)	0.0542 (7)	0.0528 (7)	−0.0212 (6)	0.0022 (5)	−0.0033 (5)
N1	0.0431 (7)	0.0386 (7)	0.0406 (7)	−0.0048 (5)	−0.0022 (5)	−0.0017 (5)
N2	0.0541 (8)	0.0445 (8)	0.0516 (8)	−0.0087 (6)	0.0033 (6)	0.0038 (6)
C1	0.0385 (8)	0.0399 (8)	0.0448 (8)	−0.0053 (6)	−0.0030 (6)	−0.0079 (6)
C2	0.0361 (8)	0.0366 (8)	0.0511 (9)	−0.0043 (6)	0.0006 (6)	−0.0085 (6)
C3	0.0473 (9)	0.0488 (9)	0.0476 (9)	−0.0087 (7)	0.0058 (7)	−0.0087 (7)
C4	0.0561 (10)	0.0465 (9)	0.0476 (9)	−0.0078 (8)	−0.0065 (7)	−0.0006 (7)
C5	0.0583 (10)	0.0432 (9)	0.0600 (11)	0.0039 (8)	−0.0119 (8)	−0.0065 (8)
C6	0.0516 (9)	0.0471 (9)	0.0509 (9)	0.0043 (7)	−0.0042 (7)	−0.0130 (7)
C7	0.0496 (9)	0.0410 (8)	0.0451 (8)	−0.0060 (7)	0.0028 (7)	−0.0059 (7)
C8	0.0407 (8)	0.0400 (8)	0.0408 (8)	−0.0041 (6)	−0.0049 (6)	−0.0005 (6)
C9	0.0600 (11)	0.0630 (12)	0.0498 (10)	0.0000 (9)	0.0086 (8)	0.0045 (8)
C10	0.0696 (12)	0.0647 (12)	0.0471 (10)	0.0031 (10)	0.0024 (8)	−0.0134 (9)
C11	0.0586 (10)	0.0470 (9)	0.0524 (10)	−0.0032 (8)	−0.0067 (8)	−0.0114 (8)

### Geometric parameters (Å, °)

**Table:**

Cl1—C2	1.7290 (15)	C4—C5	1.376 (2)
Cl2—C4	1.7362 (18)	C5—C6	1.380 (2)
O1—C1	1.3613 (18)	C7—C8	1.506 (2)
O1—C7	1.417 (2)	C9—C10	1.400 (3)
O2—C8	1.200 (2)	C10—C11	1.343 (3)
N1—N2	1.3695 (19)	C3—H3	0.9300
N1—C8	1.397 (2)	C5—H5	0.9300
N1—C11	1.363 (2)	C6—H6	0.9300
N2—C9	1.309 (2)	C7—H7A	0.9700
C1—C2	1.393 (2)	C7—H7B	0.9700
C1—C6	1.386 (2)	C9—H9	0.9300
C2—C3	1.374 (2)	C10—H10	0.9300
C3—C4	1.375 (2)	C11—H11	0.9300
			
Cl1···O1	2.8447 (12)	C2···C6^i^	3.476 (2)
Cl1···C1^i^	3.5263 (15)	C3···C5^i^	3.474 (2)
Cl1···C2^i^	3.5736 (14)	C5···C2^viii^	3.469 (2)
Cl1···Cl2^ii^	3.6862 (7)	C5···C3^viii^	3.474 (2)
Cl2···Cl1^ii^	3.6862 (7)	C6···O2	3.188 (2)
Cl1···H9^iii^	3.0200	C6···C1^viii^	3.592 (2)
Cl1···H3^iv^	3.0300	C6···C2^viii^	3.476 (2)
Cl2···H10^v^	3.1400	C6···C8	3.252 (2)
Cl2···H5^vi^	3.0100	C7···N2^iii^	3.387 (2)
O1···Cl1	2.8447 (12)	C8···N2^i^	3.313 (2)
O1···O2	2.7368 (17)	C8···C6	3.252 (2)
O2···N2^i^	3.2665 (19)	C9···C11^viii^	3.370 (3)
O2···O1	2.7368 (17)	C9···N1^viii^	3.445 (2)
O2···N1^i^	3.2466 (18)	C11···C9^i^	3.370 (3)
O2···C1	3.1942 (19)	C11···O2^vii^	3.339 (2)
O2···C6	3.188 (2)	C6···H7A	2.6600
O2···C11^vii^	3.339 (2)	C7···H6	2.6200
O1···H7A^i^	2.6100	C8···H6	2.7200
O2···H6	2.7500	H3···Cl1^iv^	3.0300
O2···H11	2.7700	H5···Cl2^vi^	3.0100
O2···H11^vii^	2.4200	H6···O2	2.7500
N1···O2^viii^	3.2466 (18)	H6···C7	2.6200
N1···C9^i^	3.445 (2)	H6···C8	2.7200
N2···O2^viii^	3.2665 (19)	H6···H7A	2.3000
N2···C8^viii^	3.313 (2)	H7A···O1^viii^	2.6100
N2···C7^iii^	3.387 (2)	H7A···N2	2.7700
N2···H7A	2.7700	H7A···C6	2.6600
N2···H7B	2.4800	H7A···H6	2.3000
N2···H7B^iii^	2.7000	H7B···N2	2.4800
C1···Cl1^viii^	3.5263 (14)	H7B···N2^iii^	2.7000
C1···O2	3.1942 (19)	H9···Cl1^iii^	3.0200
C1···C6^i^	3.592 (2)	H10···Cl2^ix^	3.1400
C2···Cl1^viii^	3.5736 (14)	H11···O2	2.7700
C2···C5^i^	3.469 (2)	H11···O2^vii^	2.4200
			
C1—O1—C7	118.85 (12)	N2—C9—C10	112.42 (17)
N2—N1—C8	120.45 (13)	C9—C10—C11	105.58 (16)
N2—N1—C11	111.72 (13)	N1—C11—C10	106.55 (16)
C8—N1—C11	127.80 (13)	C2—C3—H3	121.00
N1—N2—C9	103.74 (14)	C4—C3—H3	121.00
O1—C1—C2	116.28 (13)	C4—C5—H5	120.00
O1—C1—C6	125.34 (14)	C6—C5—H5	120.00
C2—C1—C6	118.38 (14)	C1—C6—H6	120.00
Cl1—C2—C1	118.80 (12)	C5—C6—H6	120.00
Cl1—C2—C3	119.68 (12)	O1—C7—H7A	109.00
C1—C2—C3	121.49 (14)	O1—C7—H7B	109.00
C2—C3—C4	118.88 (15)	C8—C7—H7A	109.00
Cl2—C4—C3	119.31 (13)	C8—C7—H7B	109.00
Cl2—C4—C5	119.64 (14)	H7A—C7—H7B	108.00
C3—C4—C5	121.04 (16)	N2—C9—H9	124.00
C4—C5—C6	119.73 (16)	C10—C9—H9	124.00
C1—C6—C5	120.47 (16)	C9—C10—H10	127.00
O1—C7—C8	111.44 (13)	C11—C10—H10	127.00
O2—C8—N1	121.06 (14)	N1—C11—H11	127.00
O2—C8—C7	124.89 (14)	C10—C11—H11	127.00
N1—C8—C7	114.05 (13)		
			
C7—O1—C1—C2	160.01 (13)	C6—C1—C2—C3	−0.8 (2)
C7—O1—C1—C6	−20.0 (2)	O1—C1—C6—C5	−179.24 (15)
C1—O1—C7—C8	85.03 (17)	C2—C1—C6—C5	0.8 (2)
C8—N1—N2—C9	−177.90 (15)	Cl1—C2—C3—C4	−178.20 (13)
C11—N1—N2—C9	0.21 (18)	C1—C2—C3—C4	0.0 (2)
N2—N1—C8—O2	174.44 (14)	C2—C3—C4—Cl2	179.37 (12)
N2—N1—C8—C7	−6.0 (2)	C2—C3—C4—C5	0.8 (3)
C11—N1—C8—O2	−3.3 (3)	Cl2—C4—C5—C6	−179.41 (14)
C11—N1—C8—C7	176.21 (15)	C3—C4—C5—C6	−0.9 (3)
N2—N1—C11—C10	−0.18 (19)	C4—C5—C6—C1	0.0 (3)
C8—N1—C11—C10	177.76 (16)	O1—C7—C8—O2	−9.1 (2)
N1—N2—C9—C10	−0.2 (2)	O1—C7—C8—N1	171.34 (13)
O1—C1—C2—Cl1	−2.56 (18)	N2—C9—C10—C11	0.1 (2)
O1—C1—C2—C3	179.19 (14)	C9—C10—C11—N1	0.1 (2)
C6—C1—C2—Cl1	177.44 (12)		

Symmetry codes: (i) *x*−1, *y*, *z*; (ii) −*x*+1, −*y*+2, −*z*+1; (iii) −*x*+2, −*y*+2, −*z*; (iv) −*x*, −*y*+2, −*z*+1; (v) *x*−1, *y*, *z*+1; (vi) −*x*+2, −*y*+1, −*z*+1; (vii) −*x*+1, −*y*+1, −*z*; (viii) *x*+1, *y*, *z*; (ix) *x*+1, *y*, *z*−1.

### Hydrogen-bond geometry (Å, °)

**Table:**

*D*—H···*A*	*D*—H	H···*A*	*D*···*A*	*D*—H···*A*
C11—H11···O2^vii^	0.93	2.42	3.339 (2)	170

Symmetry codes: (vii) −*x*+1, −*y*+1, −*z*.
